# An application of PRECIS-2 to evaluate trial design in a pilot cluster randomised controlled trial of a community-based smoking cessation intervention for women living in disadvantaged areas of Ireland

**DOI:** 10.1186/s40814-022-00969-6

**Published:** 2022-01-25

**Authors:** Catherine Darker, Kirsty Loudon, Nicola O’Connell, Stefania Castello, Emma Burke, Joanne Vance, Caitriona Reynolds, Aine Buggy, Nadine Dougall, Pauline Williams, Fiona Dobbie, Linda Bauld, Catherine B. Hayes

**Affiliations:** 1 Public Health & Primary Care, Institute of Population Health, School of Medicine, Trinity College Dublin, Dublin, Ireland; 2 Freelance Researcher, Edinburgh, UK; 3 Irish Cancer Society, Dublin, Ireland; 4 Health Promotion and Improvement, Health Service Executive, Dublin, Ireland; 5 School of Health & Social Care, Edinburgh Napier University, Edinburgh, Scotland; 6 Public and Patient Representative, Dublin, Ireland; 7 Usher Institute and SPECTRUM Consortium, College of Medicine, University of Edinburgh, Edinburgh, Scotland

**Keywords:** PRECIS-2, Pragmatic trial, Process evaluation, Implementation, Smoking cessation, Women, Deprivation, Pilot and feasibility study, Trial design

## Abstract

**Background:**

“We Can Quit2” (WCQ2) was a pilot cluster randomised controlled trial with an embedded process evaluation assessing the feasibility and acceptability of ‘We Can Quit’ (WCQ, a peer-delivered community-based stop-smoking programme for women in disadvantaged communities. The control group comprised ‘enhanced usual care’ offered by the Irish Health Service Executive (HSE). The PRagmatic Explanatory Continuum Indicator Summary (PRECIS-2) is a tool to assess whether a trial design is more explanatory (working under ideal conditions) or pragmatic (working under ‘real-world’ conditions). The aim of this paper was to retrospectively evaluate the WCQ2 pilot trial using PRECIS-2 to inform the decision-making process on progression to a future definitive trial (DT).

**Methods:**

The WCQ2 trial protocol and HSE standard stop-smoking service were described across the nine PRECIS-2 domains: eligibility, recruitment, setting, organisation, flexibility-delivery, flexibility-adherence, follow-up and primary outcome. Team members scored the domains as pragmatic or explanatory for each arm in a half-day workshop.

**Results:**

Seven team members (practitioners and researchers) assessed the overall trial design as more explanatory than pragmatic. Important differences emerged between the two arms. WCQ targeted adult women from disadvantaged communities whereas HSE run a limited enhanced service for all quitters. Trial recruitment was challenging, intense efforts were needed as the trial proceeded. WCQ was delivered in a non-clinical community setting, HSE services in a clinical setting. WCQ organisation was co-designed with community partners and comprises peer-to-peer group support delivered by trained lay community facilitators, whereas HSE one-to-one support is delivered by Smoking Cessation Officers with a clinical background. Only WCQ allowed flexibility in delivery and adherence. Follow-up was more intensive in WCQ. Greater efforts to improve participant retention will be required in a future DT.

**Conclusions:**

PRECIS-2 allowed the reflection of practitioners and researchers on similarities and differences between intervention and control arms. Results will inform the decision on progression to an effectiveness DT, which will require more a pragmatic and less explanatory design. This novel use of PRECIS-2 to retrospectively evaluate a complex community-based pilot trial in advance of a full DT will also support learning for those undertaking hybrid trials of implementation and effectiveness.

**Trial registration:**

This trial is registered with the ISRCTN registry (No. 74721694).

**Supplementary Information:**

The online version contains supplementary material available at 10.1186/s40814-022-00969-6.

## Key messages regarding feasibility

1) What uncertainties existed regarding the feasibility?

- The PRECIS-2 tool is usually used prospectively in planning trial designs. Retrospective application to a pilot trial as a part of a process evaluation is a novel application.

2) What are the key feasibility findings?

- The application of the PRECIS-2 tool by a multidisciplinary team of researchers and practitioners to retrospectively evaluate the We Can Quit2 pilot trial in terms of pragmatic and explanatory characteristics showed that the trial design was more explanatory than pragmatic.

3) What are the implications of the feasibility findings for the design of the main study?

- Results will inform the decision on progression to a full definitive trial, which will require a more a pragmatic and less explanatory design. They will also support learning for those undertaking future hybrid trials of implementation and effectiveness.

## Background

Worldwide, tobacco use causes more than 7 million deaths per year, and if this pattern remains unchanged, more than 8 million people a year will die from diseases related to tobacco use by 2030 [1]. In Ireland, almost 6000 smokers die each year from smoking-related diseases [2]. Despite a decline in the prevalence of smoking from 23% in 2015 to 17% in 2019, 14% of Irish adults identify themselves as daily smokers [3]. There is substantial evidence that people living in poverty carry the heaviest burden of tobacco related premature death and disability [4]. This is also true in Ireland, where higher rates of smoking are more likely to occur in more deprived areas (24%), compared to more affluent areas (14%) [3]. Research has also demonstrated gender-specific effects in smoking cessation. Women are less likely to achieve smoking abstinence than men [5]. There are also differences in terms of smoking cessation treatment needs. Taking nicotine replacement therapy (NRT) in conjunction with high-intensity non-pharmacological support is more effective for women than men [6]. NRT and low support were effective for women only at short-term follow-up, whereas men benefited from NRT at all the follow-ups regardless of the intensity of the adjunct support. The results suggested that long-term maintenance of NRT treatment gains decrease more rapidly for women than men [6]. A recent review of gender based differences in smoking cessation contended that women have more difficulty in achieving longer term abstinence from smoking than men [5]. As reflected internationally, 46% of smokers in Ireland reported a quit attempt in the past 12 months, and 28% have been either trying to quit or planning to do so [3]. Capitalizing on this ‘readiness to quit’ is a core feature of many smoking cessation programmes.

‘We Can Quit’ (WCQ) is a peer-delivered community-based smoking cessation programme for women smokers from socioeconomically disadvantaged (SED) areas. It was developed by the Irish Cancer Society (ICS), Ireland’s largest cancer charity, in partnership with the National Women’s Council of Ireland, the Institute of Public Health in Ireland and the Health Service Executive (HSE) [7]. Key elements are based on the ‘Sister to Sister’ programme in the USA [8, 9]. WCQ comprises peer-support group sessions delivered in a community setting, including a combination of behavioural change techniques to enhance readiness to quit, improve self-efficacy and relapse prevention, and access to combination NRT, delivered over a 12-week period. Following a small single-arm feasibility study [7], WCQ was tested in a pilot randomised controlled trial (RCT), ‘We Can Quit 2’ (WCQ2) [10]. A process evaluation was embedded in the pilot trial to test the robustness of trial design with respect to delivery of the intervention, implementation processes and key mechanisms of impact, from which to facilitate progression to a full definitive trial.

Explanatory randomised controlled trials (RCTs) are undertaken in optimal conditions to determine efficacy of interventions, however, the applicability of their results may be limited [11]. Pragmatic RCTs maximise the future applicability of results to usual care settings by informing real-world decisions of policymakers, clinicians and patients [12]. There is a continuum rather than a dichotomy between explanatory and pragmatic trials. PRECIS-2 (Pragmatic Explanatory Continuum Indicator Summary) is a tool to assist those involved in multi-disciplinary trial design: trialists, health professionals and patient representatives, to assess where in this continuum the trial design is placed to ensure it aligns with the desired purpose [12, 13]. PRECIS-2 highlights when a trial design does not match real-world conditions. To date the PRECIS-2 tool has been mainly used to assist in design of a wide range of international definitive trials in service settings, including palliative care services [14] and health promotion interventions [15–17].

The WCQ2 pilot study was intended to be a pragmatic rather than an explanatory trial [10], in that the design choices aimed to be as close as possible to the real-world conditions of smoking cessation services usually delivered in Ireland. In our study, a retrospective evaluation of the WCQ2 pilot trial using PRECIS-2 was conducted to inform the decision-making process on progression to a future definitive trial (DT). The key components of the WCQ intervention and control treatment in real-world community health settings were described through the nine PRECIS-2 domains to assist in evaluation of the trial design along the pragmatic versus explanatory continuum. The WCQ2 trial team members were guided to carefully consider the domains of PRECIS-2 for assessment of applicability of trial design [10]. The components of the process evaluation were guided by these domains. While the vast majority of trials use PRECIS-2 as a planning tool for full definitive trials [18], the few that have used the tool at the pilot and feasibility stage have not integrated the tool framework into a process evaluation [19]. To our knowledge, WCQ2 is the first study which aimed to retrospectively apply PRECIS-2 in a pilot cluster RCT as a part of the trial process evaluation.

## Methods

### WCQ2 trial overview

WCQ2 was designed as a pilot feasibility cluster RCT seeking to test the feasibility and acceptability of the WCQ programme and trial-related features (e.g. randomisation, recruitment, data collection methods), data quality and completion rates at 12 weeks and at 6 months, and to estimate sample size and appropriateness of design for a future DT. The WCQ2 protocol has been previously published [10]. Briefly, the pilot trial was conducted in four consecutive waves in partnership with the HSE and the ICS. Each wave iteratively improved the recruitment strategy protocol, with the final wave successfully achieving the expected recruitment rate.

The intervention arm comprised the WCQ programme the control arm the HSE’s ‘enhanced usual care’ smoking cessation service, a one-to-one service delivered by a specialist smoking cessation professional. The first session was delivered face-to-face, with an option for telephone-based follow-up calls, over six to seven sessions.

Results from the trial recently published [20], indicated the feasibility and overall acceptability of conducting WCQ in a community setting and constituted valuable data to enhance the design of a future DT to assess the effectiveness of a community-based smoking cessation intervention for women living in SED areas.

### Description of PRECIS-2

The first three domains ‘eligibility’, ‘recruitment’ and ‘setting’ describe who is included in the trial and where it is carried out. The next three domains, ‘organisation’, ‘flexibility in delivery’ and ‘flexibility in adherence’ describe the intervention, what expertise and resources were put into delivering it and what steps are taken to ensure the participants in the trial and the people delivering the intervention adhere to the protocol. The final three domains, ‘follow up’, ‘primary outcome’ and ‘primary analysis’ describe the data from the trial, what and when it collected and how is it analysed. PRECIS-2 has been found to have good interrater reliability and moderate discriminant validity [18].

To apply PRECIS-2, a detailed description of each domain is collated and each domain is scored from 1 to 5 using a 5-point Likert scale (where 1 indicates a very explanatory design, testing an intervention under ideal conditions and 5 a very pragmatic design, replicating usual care conditions for that domain. Once scores have been allocated to the nine domains, a PRECIS-2 wheel may be plotted for the trial, highlighting design aspects of the trial that are closer to usual care and those which are not. Researchers may then consider whether or not the design matches the purpose of the trial; in the case of an explanatory trial, a more tightly controlled trial under ideal conditions, that aims to provide understanding of how treatments work; in the case of a pragmatic trial, producing relevant results that can influence clinical practice and be applied to improve healthcare. The Health Research Board in Ireland, which funded the WCQ2 trial, encourages trialists to use PRECIS-2 in their guidance documents [21].

### Procedure

A half-day workshop was convened for the WCQ2 trial team (seven individuals) to facilitate use of the PRECIS-2 tool to assess the pragmatism of the pilot feasibility study design. The trial team planned to conduct the workshop in September 2019, when trial data collection was complete, after the final wave of the trial, to inform decisions on whether and how to proceed to a definitive RCT. Participants included one HSE member of staff, two non-governmental organisation partners including the ICS and four WCQ2 staff (primary investigator (PI), trialist focussing on process evaluation, research fellow and research assistant).

### Pre-meeting training

The WCQ2 trial team provided descriptive information on the WCQ (intervention arm) and enhanced usual care (control arm) mapped to the nine domains of PRECIS-2 (see Additional file 1), in the weeks prior to the workshop. Information on WCQ and the HSE standard smoking cessation programmes under real-world conditions was also reviewed against the PRECIS-2 domains and shared with the team beforehand as a part of a description document (Additional file 1). A draft version of this document was circulated amongst workshop participants for inputs and comments in advance of the meeting.

The WCQ2 team were given registration details to access the PRECIS-2 website www.PRECIS-2.org to use software to create their own PRECIS-2 study wheel. Individuals were also sent copies of the BMJ elaboration paper for PRECIS-2 [13], and an information sheet to assist in using the PRECIS-2 wheel to score WCQ2. Participants were encouraged to ask questions on using the tool.

### Half-day workshop

At the meeting, handouts with the WCQ2 PRECIS-2 wheel including domain scores and scoring rationale for each participant were used to facilitate discussion. The draft description document included details of the WCQ intervention and the standard HSE smoking cessation services, and a description of the trial protocol [10] for each of the PRECIS-2 domains.

The half-day workshop was facilitated by the original PRECIS-2 lead author (KL) to assist in scoring the domains of the tool. Each participant attending the workshop scored each domain independently and then a facilitated discussion ensued which allowed individuals to describe the rationale for their score. Members of the workshop were given an opportunity to change their score after each individual had given their viewpoint. The PRECIS-2 tool was used to discuss the appropriateness of the trial design, not to measure changes in scores; hence, the methodology was more qualitative than quantitative. This was a novel application of PRECIS-2 for a pilot and feasibility study.

## Results

The results of the PRECIS-2 domain assessments indicating how WCQ2’s original implementation strategy mapped onto the pragmatic-explanatory continuum are detailed in Additional file 1. The facilitated discussion clarified the content describing the WCQ intervention and enhanced usual care arms. Most of the work for this had been undertaken earlier through the embedded process evaluation and open sharing of information by the trial team.

### Overall PRECIS-2 scoring

**Fig. 1:**
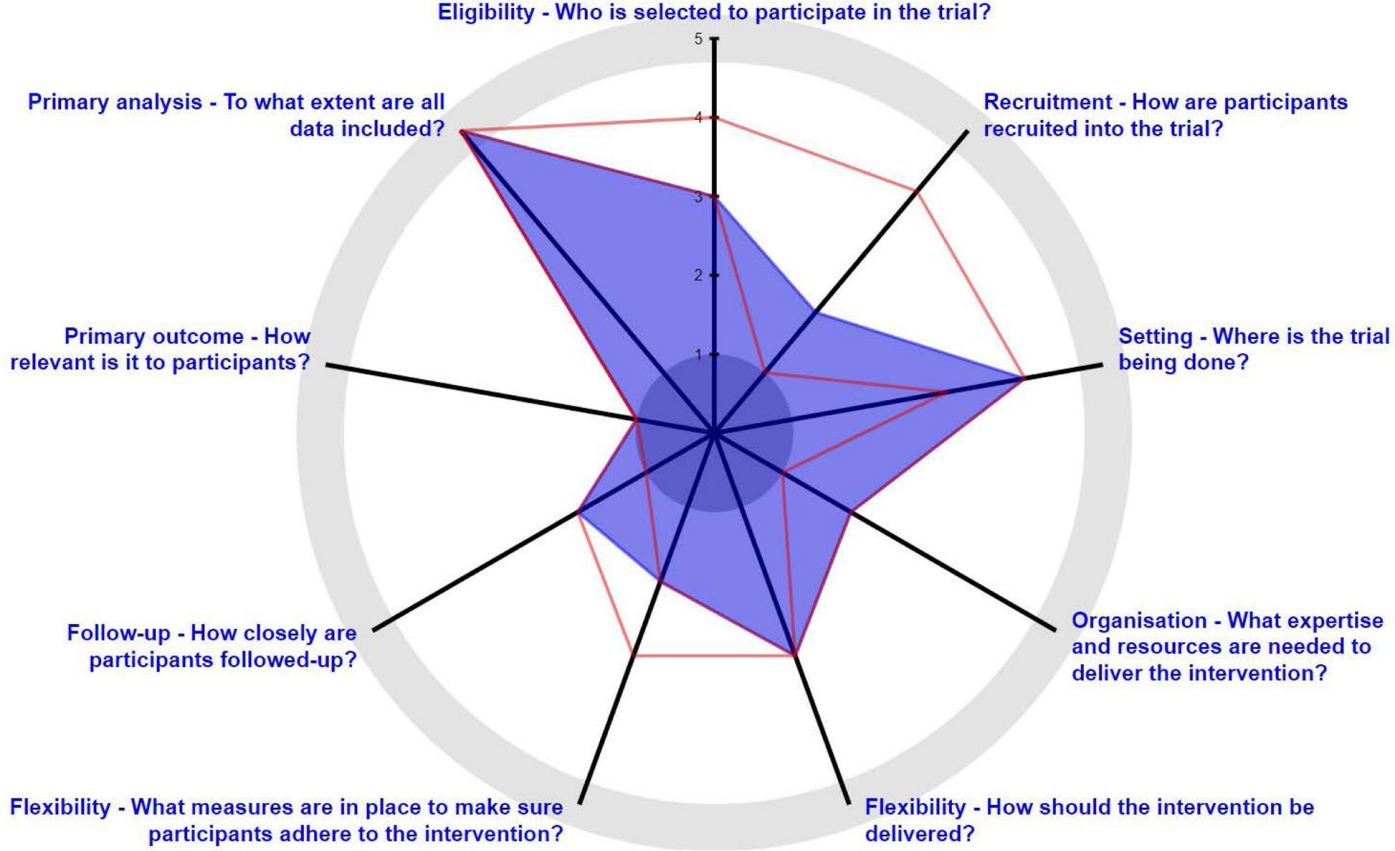
Composite score PRECIS-2 wheel for seven WCQ2 team members

The seven team members assessed the overall design of the WCQ2 feasibility study as more explanatory than pragmatic (Fig. 1, Table 1). There were two domains with consensus: (1) “primary outcome” and (2) “flexibility of adherence” (of the intervention). The primary analysis domain was not scored by the team as that had been pre-determined as an intention to-treat analysis by previous discussions involving the WCQ2 trial statistician and was therefore deemed not relevant for the purposes of PRECIS-2. There was, however, no more than one point difference in scores (out of 5) for seven domains, suggesting there was little difference in rating domains. Recruitment has the widest range from “1-4”, with 3 scores of “1”, 3 scores of “2” and an outlier of "4".

**Table 1:** Postworkshop scores for PRECIS-2 domains for seven WCQ2 team members^a^

PRECIS-2 domains	Trial PI	Trialist—process evaluation	Research fellow	Research assistant	NGO partner	NGO partner	HSE staff
***Eligibility***	3	4	3	3	3	3	3
***Recruitment***	2	2	4	2	1	1	1
***Setting***	4	4	4	4	3	4	4
***Organisation***	1	1	1	2	2	2	2
***Flex delivery***	3	3	3	3	3	3	3
***Flex adherence***	2	2	2	2	3	2	2
***Follow up***	2	1	2	2	1	1	2
***1ry outcome***	1	1	1	1	1	1	1
***1ry analysis***	N/A						

^a^Lay person/public patient involvement absent from the workshop but involved in pre-meeting activities

*PI* Primary investigator, *NGO* Non-governmental organisation, *HSE* Health service executive

### Rationale of PRECIS-2 scoring for WCQ2

#### Eligibility (median score 3)

WCQ2 trial targeted women aged over 18 in specific socio-economic areas who spoke English, whereas HSE standard services typically target both men and women of any age, who speak any language and live in an area in which a smoking cessation officer is available.

#### Recruitment (median score 2)

WCQ2 recruitment strategies were more diverse and intense than usual HSE strategies. Local Advisory Groups (LAGs) were established in each of the study areas. These included local people from the areas who would have an established role in community development. The role of the LAG was to oversee WCQ2 trial conduct, and to direct and deliver a local recruitment strategy. WCQ2 developed a recruitment strategy which included community stakeholders to undertake recruitment with tailor-made leaflets, posters, flyers, and Facebook posts. The PI established contact with local general practitioners who were encouraged to actively recruit, as were local pharmacists. There was also a designated person from the ICS within each study area whose role included assisting and coordinating recruitment. In addition, a WCQ2 trial member was assigned for recruitment. The HSE usual recruitment routes include referral by healthcare provider, self-referral, referral through a national quit team/online support, and on-going national quit campaigns that occur through mediums such as TV, radio and cinema advertisements. During the final wave of recruitment, an adaptation of the recruitment protocol introduced paid social media advertising, leaning towards a more pragmatic approach.

#### Setting (median score 4)

WCQ was community based, typically taking place in a community centre, whereas the enhanced usual care sessions were delivered in a clinical setting such as a hospital or a primary care centre. WCQ2 focussed on delivery of the programme within deprived communities, and typically these areas were also within the catchment areas of HSE standard services. The selection of areas within the pilot trial was limited to areas where the HSE had a smoking cessation advisor available to deliver their one-to-one service.

#### Organisation (median score 2)

WCQ organisation was very different from the HSE standard organisation format. WCQ has been co-designed in partnership with the community. The outcome of each programme was celebrated and shared with the community. Participants were encouraged to share their experience with others.

The delivery format was group-based. WCQ offered face-to-face peer support groups for women, involving trained Community Facilitators working in pairs (one was ideally an ex-smoker). Many were specifically recruited for this smoking cessation programme. The programme delivery applied a social model of health to tobacco cessation. Community Facilitators were trained in both the National Standard for Tobacco Cessation Support (NSTCS) [22] and the Social Determinants of Health framework including issues of gender, income, health access, self-efficacy and educational inequality to their content and program delivery. Some were experienced group facilitators; others were also trained in group facilitation. The HSE Smoking Cessation Officers are trained to offer behavioural support and advice relating to quitting smoking and maintenance on a one-to-one basis. This is also based on the NSTCS. Most Smoking Cessation Officers have clinical backgrounds (e.g. nursing). The HSE operate a nationally available ‘Quit Line’ offering free telephone and text support. The control arm of the trial, therefore, could be considered ‘enhanced’ usual care.

#### Intervention flexibility delivery (score 3)

The WCQ programme structure allowed for tailoring and flexibility in programme delivery. Within the last 6 of 12 sessions women were encouraged to choose content and activities based on a menu of locally available options that they considered would be of benefit to their quit attempt (e.g. practical healthy eating and physical activity workshops; additional stress management; women’s health or relaxation workshops). This degree of flexibility or choice was not available within the enhanced usual care control arm. Therefore, there was variation between the intervention and control arms in relation to the number, content and length of sessions. WCQ community facilitators worked with pharmacists to assist women to get access to NRT. There was self-monitoring by community facilitators through the use of a diary and checklist to monitor treatment fidelity regarding programme delivery, which were returned to the trial team.

#### Flexibility adherence (median score 2)

In our trial, the WCQ community facilitators used verbal encouragement during the face-to-face sessions and email, text and phone support between sessions to promote participation. WCQ women were never excluded if they missed sessions, they were always welcome to attend. Women enrolled into WCQ were allocated a designated local community pharmacist to dispense their NRT. Women reported that they would often seek additional support from the pharmacist between meetings. Pharmacists also encouraged continuation of attendance at weekly programme meetings. In the control arm women were not formally linked to a community pharmacist.

An incentive of €20 voucher to complete data at the 12-week and 6-month follow-ups was provided to all trial participants.

#### Follow-up (median score 2)

WCQ was longer in duration than the control arm. WCQ women attended weekly sessions for 12 weeks, whereas in enhanced usual care arm women were seen for between 6 and 8 sessions. Retention, number of sessions attended, engagement in smoking cessation processes (e.g. setting a quit date) and completion of data were closely monitored in each arm. Corroborated smoking abstinence rates at 12 weeks and at 6 months were assessed. Both groups completed the 12-item Short Form Survey questionnaire (SF-12) [23] to measure function and well-being status across each study data collection time point (baseline, 12 weeks and 6 months). Qualitative interviews were conducted after week 12 in the WCQ arm with intervention women and the community facilitators who delivered the intervention. Community facilitators were also asked to complete measures relating to acceptability, appropriateness and feasibility [24]. Interviews were not conducted with control arm participants.

#### Primary outcome (score 1)

As this was a pilot and feasibility study, the main outcomes for the trial were recruitment and retention as part of feasibility testing. All workshop participants were in agreement that “stopping smoking” would be the very pragmatic primary endpoint, scoring “5”, in the full DT, for women, for healthcare providers and from a public health perspective.

## Discussion

PRECIS-2 aims to make explicit the impact that design choices will have on the relevance of trial results to the users beyond trial conditions. The PRECIS-2 tool was originally designed to be used prospectively, as a planning tool for the design of RCTs. It has been also used retrospectively as a tool to assess the pragmatic or explanatory characteristics of RCTs in systematic reviews [25, 26] or to assess trials that were already in progress [18, 27]. To our knowledge, this is the first study which describes the retrospective use of PRECIS-2 in a pilot cluster RCT as a part of the trial process evaluation. Our results indicated that the overall WCQ2 pilot trial design was more explanatory than pragmatic, contrary to the overall intended purpose of the trial. Characteristics of recruitment, organisation, flexibility in adherence and follow-up of the pilot study design were scored as explanatory domains, and primary outcome as very explanatory. The setting of WCQ2 study in the community was the most pragmatic characteristic of the design. Eligibility and flexibility in delivery were assessed as equally pragmatic/explanatory domains.

Few pilot and feasibility studies have been published using the PRECIS-2 tool in their design.

A recently published pilot trial of a surgical intervention, the pGO-Tibia pilot in Tanzania [28], contained both pragmatic and explanatory aspects but ultimately tended towards a pragmatic design to facilitate implementation in their chosen settings. Similar to our pilot trial, the main outcomes were recruitment and retention [29], which resulted in the Primary outcome domain assessment as very explanatory which is to be expected. In a future DT of the effectiveness of WCQ2 the primary outcome will be smoking abstinence, matching the real-world environment.

The domain matrix (Additional file 1) was the basis for discussion on design improvements for the future definitive RCT. Recruitment rates were closely monitored throughout the four trial waves. Intense monitoring of recruitment efforts led to advancements made on the recruitment strategy to maximise recruitment, which resulted in this domain becoming very explanatory. These additional recruitment efforts, while effective and necessary to reach target numbers in the final recruitment wave, may have introduced a selection bias [30] in the eligibility criteria. A pragmatic approach would recruit women smokers who present themselves to routine care for assistance with smoking cessation, whereas WCQ2 used additional recruitment methods as well as encouraging participants to bring friends. We believe this was unavoidable under trial conditions.

Retention was also challenging, and in a DT more intensive efforts would be required to assist participants to complete follow up [31]. The ‘organisation’ of a DT would also require changes to incorporate both the community facilitators’ and researcher specific training in working with women who have low literacy. This was a major barrier to recruitment and retention in the pilot trial. More support to complete data collection and a greater adaptation of data forms will also be needed in a future trial. This may improve the accessibility of programme resources and trial documentation for the targeted groups of women who were trying to stop smoking. In terms of ‘flexibility of delivery’ of the intervention, the WCQ2 team recommended more structured contact with women between sessions. However, objective measurements for monitoring of the fidelity of intervention delivery would still be advocated.

The WCQ2 trial team found that PRECIS-2 may be useful to capture trial design discussion from inception to the definitive RCT [32]. The key to using PRECIS-2 was an in-depth knowledge of standard practice to stop smoking in Ireland; local expert knowledge was important to complete the PRECIS-2 domains and assist the trial team to determine the gap between the trial intervention and usual care. In this study, which focussed on supporting women living in deprived areas to stop smoking, this information is critical to facilitate further implementation of the programme into a full-scale trial in similar areas. Lack of a clear description of the usual care comparator in trials has previously been highlighted [33], with clear reporting being encouraged to ensure adherence to the CONSORT guidelines [34, 35]. The WCQ2 team endeavoured to provide an in-depth description of both the intervention and the comparator, and the PRECIS-2 tool guided this information sharing. It provided a framework for a shared understanding amongst public health academics, health promotion practitioners and public/patient representatives of the key components of both interventions and their key differences as well as their strengths and weaknesses for their target audiences.

We believe that a strength of this study is the detailed descriptive information from the trial team (Additional file 1) which concurs with others using the tool to assist in generalisability of findings [36]. Our domain information, however, is specific to this WCQ2 pilot study and may not necessarily be generalisable to other settings. It is worth emphasising that the rationale behind the PRECIS-2 score is most important for implementation, rather than the score in itself. Our discussions also highlighted an issue with enhanced usual smoking cessation programmes in the target areas for the HSE control arm which are not as yet universal across Ireland, which resulted in a lively debate on implementation during the pilot study.

Three limitations are worth noting. Firstly, the assessment of PRECIS-2 domains was completed by those running the trial or delivering components of the programme; therefore, there is the potential for assessment bias although presence of the author of PRECIS-2 (KL) who facilitated and guided discussion may have counter-acted this. Secondly, the PRECIS-2 assessment evaluated the fourth and final wave of the trial when recruitment challenges had been largely understood and ameliorated, and when the expected recruitment rate per wave had been achieved. Thirdly, although the patient representative, (PW) was unable to attend the PRECIS-2 meeting, her input, was sought throughout the study and she was involved in pre-meeting activities using PRECIS-2, and in reviewing this paper. This was a unique study with a ‘hands on’ approach, so we are confident through the process evaluation work and input from all at the meeting that trial participant views were represented across the stakeholders involved in the trial.

The National Institutes of Health (NIH) Pragmatic Trials Collaborative Project in the United States have introduced a trial planning and implementation project in response to an NIH Request for Applications to fund low-cost, pragmatic, patient-centred clinical RCTs. Results from this project reported that PRECIS-2 was useful in “framing the conversation” about trial design in the conduct of the feasibility studies, and finalisation of trial protocols [37]. This was also recently discussed by the National Institute on Aging for pilot stage clinical trials, which suggested that the PRECIS-2 tool could be used to optimise recruitment strategies, intervention flexibility and adherence measures in embedded pragmatic clinical trials, testing interventions to help elderly dementia patients in real-world settings [38]. The current WCQ2 project concurs with that assessment but also found PRECIS-2 useful for all domains assessed.

The learning points from applying PRECIS-2 to the WCQ2 trial may be useful for other researchers planning to conduct future trials. We believe that widening the circle of participants using PRECIS-2 to include all members of trials teams (new and experienced trialists) would facilitate discussion of all aspects of the design of a pilot RCT. Participation might include greater involvement of patient/public representatives (with appropriate training) and involvement of the steering group to bring richer perspectives from informed voices who have a thorough understanding of trial process rather than relying fully on the scores. While PRECIS-2 was only used after the final wave of recruitment in our pilot RCT, we believe it might also have been helpful to inform each wave in a dynamic iterative way and to inform adaptive trial designs.

Our findings provide further insight to assist trial teams in designing future trials of complex health promotion interventions in community settings and the learning from applying the PRECIS-2 tools to the findings of pilot and feasibility trials. To this end, PRECIS-2 should be applied in advance of definitive trial design to open up discussions on the implementation of complex trials in community settings. This important assessment stage could prevent problems and avoid clinical trial research waste (see http://researchwaste.net).

## Conclusions

PRECIS-2 enabled meaningful discussion within the trial team of the key elements of a future definitive intervention trial design, thereby improving our understanding of the applicability of trial results to assist women in deprived areas in Ireland to stop smoking. In particular, it helped the trialists consider the consequences of design decisions for WCQ2 and the gap between the WCQ intervention and the enhanced usual care control arm for smoking cessation provided by the HSE. PRECIS-2 was an important tool to support the decision on whether to undertake a full trial but only as part of the overall assessment, which included quantitative indicators of the direction of effect as well as the qualitative findings from the process evaluation, all three of which supported this decision.

## Supplementary Information

**Additional file 1.** PRECIS-2 scores for WCQ (intervention) versus HSE usual care (control).

## Data Availability

The dataset (descriptions of the WCQ2 trial arms, the HSE standard smoking cessation services, and the scoring rationale for each domain used to apply the PRECIS-2 tool) are included within the article and its Additional file 1.

## Abbreviations

NRT: Nicotine replacement therapy
WCQ: We Can Quit
SED: Socioeconomically disadvantaged
ICS: Irish Cancer Society
HSE: Health service executive
RCT: Randomised controlled trial
WCQ2: We Can Quit2
PRECIS-2: Pragmatic Explanatory Continuum Indicator Summary
PI: Principal investigator
LAGs: Local advisory groups
NSTCS: National Standard for Tobacco Cessation Support
SF-12: 12-item Short Form Survey questionnaire
NIH: National Institutes of Health
