# Supplementary material for: An application of PRECIS-2 to evaluate trial design in a pilot cluster randomised controlled trial of a community-based smoking cessation intervention for women living in disadvantaged areas of Ireland
Source: Pilot Feasibility Stud. 2022 Jan 25;8:19. doi: 10.1186/s40814-022-00969-6 (PMC8787878; doi:10.1186/s40814-022-00969-6)
Supplement: Supplementary file 1 — Additional file 1. PRECIS-2 scores for WCQ (intervention) versus HSE usual care (control). [file 40814_2022_969_MOESM1_ESM.docx]

**Additional file 1: PRECIS-2 scores for WCQ (intervention) versus HSE usual care (control).**

**Background:**

There are 9 domains within the PRECIS-2 Wheel. Within each domain there is a related description for the We Can Quit Programme (WCQ) and the Health Service Executive (HSE) Service. We have differentiated between what is Standard and what is as a result of the Trial Protocol. The purpose of this document is to clearly communicate to the reader what is Standard practice within each arm and what is as a result of implementing the Trial Protocol.

**Standard Protocol:** The “Standard Protocol” description relates to the typical activities and parameters within the WCQ Programme or HSE Service.

Note: ‘Usual care’ is defined as per trial protocol as follows: Face-to-face individual smoking cessation sessions offered by the HSE for men and women delivered by a Smoking Cessation Officer in a community setting.

**Trial Protocol:** This relates to changes to standard activities and parameters that were introduced to either the WCQ Programme or HSE Service for the purpose of running a trial in addition to the Standard Protocol criteria for the WCQ or HSE programme.

**Instructions for Scoring:**

KL will take the group through the scoring at the workshop. The continuum is on a scale of 1 to 5.

**PRECIS 5-point Likert Scale:**

1. very explanatory
2. rather explanatory
3. equally pragmatic/explanatory
4. rather pragmatic
5. very pragmatic


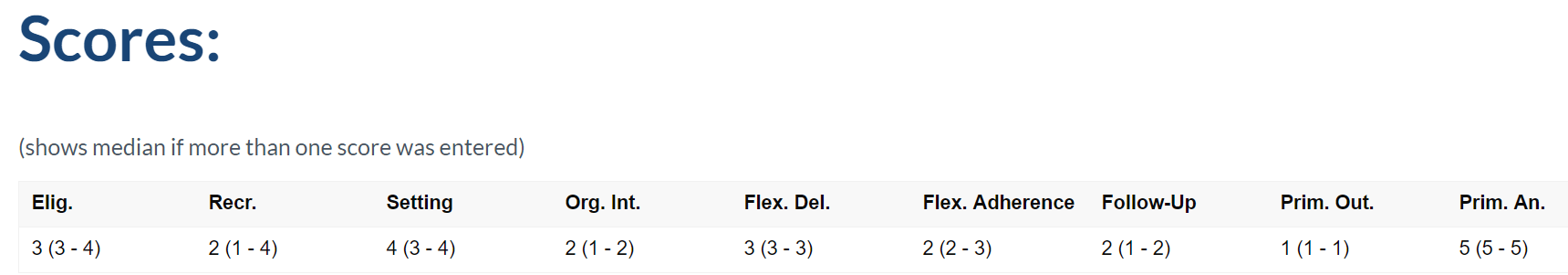


**Table 1. Description document- PRECIS-2 scores and score rationale.**

| **Domain 1**: **Eligibility criteria** | |
| --- | --- |
| *To what extent are the participants in the trial similar to those who would receive this intervention if it was part of usual care?*  **Score: 3/5**  **Rationale**:   - WCQ2 targeted women whereas HSE targets both men and women - WCQ2 treats only women over 18 whereas HSE treats any age (not many under 18 affected) - WCQ2 women in specific socio-economic areas which were deprived whereas HSE women in any area with a smoking cessation officer - WCQ2 English language speaker whereas HSE any language (some bring translator) - Also, WCQ2 women could not be pregnant or considering pregnancy in short term. This group may need more specialised support than WCQ2 programme but not key in scoring for team. | |
| ***Description of WCQ programme (Irish Cancer Society)*** | ***Description of usual care (HSE service)*** |
| - Female smokers only | - Open to both male and female smokers |
| - Over 18 years of age | - Under 18 years of age and over 18 years of age |
| - Delivery in Areas of deprivation as identified by Electoral Divisions | - Areas of country where smoking cessation officer present |
| - Understand and speak English | - Speaking any language (some bring interpreter) |
|  | - Service available to all (e.g., [www.quit.ie](http://www.quit.ie) and telephone support through HSE Quit Line). |
| ***Description of WCQ2 Trial Protocol:*** | |
| - Population size | |
| - Not pregnant or considering a pregnancy in short term | |
| - Women living and/or working within study areas and are within easy travel distance of trial catchment areas | |
| - Stringent area boundaries according to Electoral Divisions with low socio-economic-status | |
| - Daily smokers | |
| - Indicate an interest in quitting | |
| - Women taking NRT or who have been prescribed burpropion/varenicline by the doctor at the time of recruitment and women using e-cigarettes who are current smokers. | |
| - Women who have the capacity to give informed consent. | |
| - Female smokers only | |
| - Over 18 years of age | |
| - Understand and speak English | |
|  | - Location where face-to-face smoking cessation service available |
| **Domain 2: Recruitment path** | |
| *How much extra effort is made to recruit participants over and above what would be pragmatic recruitment in the usual care setting to engage with participants?*  **Score: 2/5**  **Rationale**:  Factors in WCQ2 that reduced the PRECIS-2 score:  Extra measures were put in place compared to HSE usual care to recruit women:   - WCQ2 had Facebook posts and website management by Trinity College Dublin through “Healthy Trinity” webpage. HSE is online registration. - WCQ2 specific targeting by GPs by Trial Principal Investigator via GP Unit with practitioners requested to actively recruit participants. - WCQ2 recruitment through pharmacists, as well as public health nurses and community workers who will complete online registration on their behalf. - Promotion through local community groups (e.g. childcare centres, work placement schemes) and pop ups in shopping centres, churches and creches. - Recruitment plans for each WCQ2 area developed by community stakeholders and includes tailor made information leaflets/flyers/posters and with contact details and dates. HSE is more national, on-going recruitment and can include TV, radio, cinema, and Facebook on-going advertising and at all HSE locations e.g. hospitals, clinics, etc. - WCQ community workers distribute aforementioned leaflets/flyers/posters. - Member of WCQ2 research team involved in recruitment. | |
| ***Description of WCQ programme (Irish Cancer Society)*** | ***Description of usual care (HSE service)*** |
| - Community Partners from new target area, invited to form a local advisory group to support programme delivery in their area and provide a local point of contact | - Communication campaigns increase activity for referrals |
| - At least 12 weeks in advance the Local Advisory Group agrees a recruitment plan for the area, venue, target groups, time and date, and actions for distribution and engagement. | - Referral routes include: - Referral by healthcare provider - Self-referral - Quit team/online support - On-going national quit campaign- TV, radio, cinema |
| - Use of information leaflets/flyers/posters. Different local contact details of community partner included for each area |  |
| - Use of social media/use of promoted spots on Facebook with content generated by ICS |  |
| - Community workers distribute leaflets with date, time and venue, within in their services and support participants to register their interest online |  |
| - Information provided to local General Practitioners, pharmacist and primary care network co-ordinator, active involvement of Primary Care team in referring patients encouraged |  |
| - Snowballing: those who register interest are encouraged to spread the word prior to programme delivery |  |
| - Local press release (print and radio) - Pre programme Information session (usually in new target area) - Registered participants are contacted by email (Irish Cancer Society) and by phone to confirm their interest and place on the course. Written informed participant consent for communication and monitoring and evaluation purposes by Irish Cancer Society and Health Service Executive is requested at week 1 of the group on a one to one basis, by Community Facilitators or partner organisation representative. |  |
| ***Description of WCQ2 Trial Protocol:*** | |
| - Website and Facebook managed by Trinity College Dublin | |
| - Promotion boosted Facebook promotion via “Healthy Trinity” webpage | |
| - Community Facilitators share the information via Facebook and the organisations website were possible and recruit using word of mouth opportunities. | |
| - Community workers distribute leaflets in their services and support participants to register their interest online | |
| - Information provided to local General Practitioner, pharmacist and primary care network co-ordinator, active involvement of Primary Care team in referring patients encouraged | |
| - Promotion through local community groups (e.g., childcare centres, work placement schemes) pop ups in shopping centres churches, and creches | |
| - Use of information leaflets/flyers/posters | |
| - Specific targeting of GPs by Trial Principal Investigator via their GP Unit | |
| - Practitioners have been requested to actively recruit participants | |
| - General press release (print & radio) | |
| - Local press release (print & radio) | |
| - Self-referral through the project website by telephone or by email or via referral by their GP local pharmacist, public health nurse or community workers who will complete the online registration from on their behalf. | |
| - Member of research team involved in recruitment (represents additional resource) | |
| **Domain 3: Setting** | |
| *How different is the setting of the trial and the usual care setting?*  **Score: 4/5**  **Rationale**:  The setting was not a perfect match with the usual HSE delivery setting and PRECIS-2 reduced by the following factors:   - WCQ2 trial is undertaken in disadvantaged areas which is different to HSE usual care. - WCQ2 is community based whereas HSE is clinic based and some are hospital based. - WCQ2 targets towns and counties, which is similar to HSE. | |
| ***Description of WCQ programme (Irish Cancer Society)*** | ***Description of usual care (HSE service)*** |
| - Community centres (not for profit organisations who receive funding for staff and services from Department of Environment and local government under their Social Inclusion and Community Activation Programme fund, other departments within HSE) | - Takes place in the workplace of the smoking cessation officer delivering the programme e.g., health-related facilities within primary care and hospital outpatient department. |
| - Traditional areas of disadvantage | - Health Service Executive using usual settings, clinics, community facilities, primary care centres |
| - Urban city and county | - Urban, city and county |
| - Close proximity to participant’s home |  |
| - Urban, target postcodes/ housing complexes identified |  |
| ***Description of WCQ2 Trial Protocol*** | |
| - Area boundaries – 4 disadvantaged geographical districts | |
| **Domain 4: Organisation** | |
| *How different are the resources, provider expertise and organization of care delivery in the intervention arm of the trial and those available in usual care?*  **Score**: 2/5  **Rationale**:  The organisation for WCQ2 and HSE are currently very different. Potential to introduce into mainstream and building enhanced delivery capacity:   - Key difference is WCQ2 is face-to-face peer-support groups of women, attending up to 12 sessions, with facilitator and HSE is one-to-one but content quite similar. - WCQ2 uses community facilitators (work in pairs – one is ideally an ex-smoker) whereas HSE practitioners have clinical background, some psychotherapy, some nursing. WCQ2 facilitators are specifically employed for WCQ2 and are often recruited from the voluntary sector and may have had success quitting smoking themselves. - WCQ2 and HSE standardise their training according to National Standard for Tobacco Cessation Support (NSTCS) programme. WCQ gives additional course on facilitator skills as well as online level 3 NCSCT training. HSE can also be trained in Make Every Contact Count (MECC). - Different resources, WCQ2 community facilitator resource pack. - Passport to Quit celebratory moment at the end of WCQ2 programme whether or not quit smoking! No equivalent in HSE | |
| ***Description of WCQ programme (Irish Cancer Society)*** | ***Description of usual care (HSE service)*** |
| - WCQ delivery model developed and funded by the voluntary/charitable sector (Irish Cancer Society) using a partnership approach. Local course delivery is led by lead community agency, through local advisory committee, supported at national level by Irish Cancer Society and Health Service Executive who jointly train CF’s to the HSE standard in Smoking cessation (reviewed every 12 months) | - Health Service Executive is the provider of national health services. HSE programmes are therefore statutory funded services. |
| - Resources: - Irish Cancer Society grant to lead community partner to cover the basic costs of delivery (option for local advisory group to find more financial resources to run more programmes in their area) - Irish Cancer Society supply WCQ programme participant learning journal - HSE send representatives to advisory committee - Local community partners within local community centres may provide additional social supports for participants and information on access to referral pathways | - Resources: - Practitioners have variety of resources available on healthpromotion.ie and have an expertise in pathways available to participants - Local sports partnerships, Social prescribing, Mental health groups:  Pathways for people to support with other issues like loneliness, lack of activity’s, social isolation - Local community partners within local community centres may provide additional social supports for participants and information on access to referral pathways |
| - “We Can Quit” Community Facilitator Resource pack developed by Irish Cancer Society (April 2016, updated in March 2018) - Irish Cancer Society and Health Service Executive jointly train CF’s to the HSE standard in Smoking cessation (TTT programme reviewed every 12months) | - National Standard for Tobacco Cessation Support Programme (manual) - ‘Make Every Contact Count’ (MECC training) - Brief Intervention Training - Health Services Executive/National Centre for Smoking Cessation and Training online training and assessment programme - Health Services Executive/National Centre for Smoking Cessation and Training specialist modules completed - Health Services Executive/National Centre for Smoking Cessation and Training practitioners’ assessment 80% is required for pass |
| - Carbon Monoxide monitor and ‘Jar of Tar’, other props e.g. money box or stress ball, leaflets available | - Carbon Monoxide monitor |
| - Irish Cancer Society provides a staff member to support governance, monitoring and evaluation, return of smoking cessation stats to Health Service Executive Tobacco Control | - All clinical contacts are recorded on the QuitManager; HSE National Behavioural Management Support System |
| - Irish Cancer Society set up pharmacy link and manage the supply and cost of Nicotine Replacement Therapy for both GMS and non-GMS eligible women |  |
| - Irish Cancer Society manage promotion materials, website registration and promotion and local press releases and phot shoots |  |
| - Irish Cancer Society provide mentoring and support for Community Facilitators and provide refresher training sessions |  |
| - Provider expertise: Delivered by Community Facilitators. Community Facilitators are provided with 3-day training initially. Training described in “We Can Quit: Community Based Smoking Cessation Programme for Women. A Training Guide for Trainers”. Delivered jointly by Health Service Executive and Irish Cancer Society. This training is based on the National Practice Standard in Smoking cessation and provides a rationale for why and how a targeted approach to addressing tobacco related health inequalities in low income communities is used. Since January 2017 Facilitators are required to complete an online level 2 NCSCT programme at the minimum. Additional 2 day programme in facilitation skills also provided since March 2018. - New training protocol will ask every CF to do level 3 NCSCT training and MECC training | - Provider expertise: Health Service Executive Smoking Cessation Officers. - Making Every Contact Count – Brief Intervention Training, available to staff in 2018 - Two-day Health Services Executive/National Centre for Smoking Cessation and Training face to face intensive training - Training described in “Tobacco Cessation Support Programme” (March 2013). |
| - Two Community Facilitators deliver the programme in tandem |  |
| - How are the Community Facilitators selected: - Community representatives on advisory group source suitable community trainers through application form and interview process, followed by Training Needs Analysis. The model started with community stakeholders with knowledge and experience of social exclusion and who were ex-smokers. - Community Facilitators can have a background with a community remit (e.g., community development, community health, social care, community education and family support); At least one of the pair of Co-Facilitators is an ex-smoker. - Community Facilitator’s are paid for the delivery of the programme. | - Most practitioners have clinical background some with psychotherapy training. Most have nursing backgrounds |
| - Mode of delivery: As a standard, attempts are made to recruit approximately 20 to 25 women for delivery of a group of 12. However, the programme will commence delivery for a group of 8 in a new area | - Mode of delivery: One-to-one Consultation post quit date in person, by phone, by post or email/SMS at pre-defined timepoints (see Point 7) |
| - End of programme celebration event held to celebrate the achievements of the women over the course of the programme, whether this is if they have quit, cut down or wish to quit in the future. It is also to demonstrate to participants and others, the partnership between providers at community level and to support future social marketing efforts. |  |
| ***Description of WCQ2 Trial Protocol*** | |
| - All practitioners and researchers involved in our study will be trained in Good Clinical Practice. | |
| - Typically, Irish Cancer Society manage website registration but for the trial Trinity College Dublin hosted website registrations |  |
|  | - Additional day per week allocated to Smoking Cessation Officer for sessions to provide service for trial |
| **Domain 5: Flexibility – delivery** | |
| *How different is the flexibility in how the intervention is delivered and the flexibility likely in usual care?*  **Score**: 3/5  **Rationale**:  There is a WCQ2 protocol and some flexibility in delivery with core content enabling order to change with intervention delivery:   - WCQ2 tailored to individual women trying to stop smoking or cut down, based on range of options in WCQ2 protocol. - WCQ2 give text or phone support. - WCQ2 community facilitators work with pharmacists so that women get tailored Nicotine Replacement Therapy - WCQ2 there is a community facilitator diary and checklist and so passive monitoring. - There are no active monitoring measures to measure compliance of WCQ2 facilitators. If recruitment and smoking cessation rates are lower than expected, they are addressed in refresher course but not during the WCQ2 intervention period. - HSE looks at satisfaction of participants to monitor delivery of HSE programme. | |
| ***Description of WCQ programme (Irish Cancer Society)*** | ***Description of usual care (HSE service)*** |
| - Number of sessions: up to 12 sessions | - Number of sessions: between 6-7 sessions |
| - Session length: Approximately 1.5 hours | - Average session length: between 30-45 minutes |
| - Core and optional components: Yes | - Core and optional components: Yes |
| - Evening and morning depending on demand, CF availability and venue availability | - Daytime sessions. Health Service Executive run evening sessions when available |
| - Additional support includes one-to-one contact (by phone or text or F-2-F) by Community Facilitators. - Option of one-to-one weekly drop in with Pharmacist if preferred - CF checklist and diary - No supervision compliance (passive monitoring) | - Face-to-face - Telephone support - No supervision compliance (passive monitoring) |
| ***Description of WCQ2 Trial Protocol:*** | |
| - No changes were made in the delivery of either programme as a result of the trial. | |
| **Domain 6: Flexibility – adherence** | |
| *How different is the flexibility in how participants must adhere to the intervention and the flexibility likely in usual care?*  **Score**: 2/5  **Rationale**:  Factors in WCQ2 that reduced the PRECIS-2 score:   - Flexibility adherence more than just “encouragement”: - In WCQ2 women encouraged as much as possible to attend through tailored support for individual women, also texts and phone calls. - In HSE if a woman misses 2 sessions, she is excluded. If there is no response to text or email, WCQ2 participants not withdrawn if fail to attend. - In WCQ2, there is also “one-to-one” drop in with pharmacist if preferred and if a woman misses sessions. Pharmacist asks participants to contact Community Facilitators for a chat and to confirm that they are still in WCQ and are encouraged to attend group on a weekly basis | |
| ***Description of WCQ programme (Irish Cancer Society)*** | ***Description of usual care (HSE service)*** |
| - Participants are encouraged to commit to attend as many sessions as possible | - Participants agree to attend sessions |
| - Set a quit date goal | - Set a quit date goal |
| - Set about achieving that goal with 3 support factors provided, e.g. group support, one to one support from facilitator, Nicotine Replacement Therapy support from Pharmacy | - Set about achieving that goal with support provided |
| - Three follow-up time points: Week 12, 6 months and 12 months. Measurement is taken from programme start date. | - At 1, 3 and 12 months, clients are telephoned to ask about their current quit status (follow up – the date the client quits is counted as day 1) |
| - Record of attendance kept | - Record of attendance kept |
| - Community Facilitator calls/texts weekly to maintain attendance. Option for participant to call the facilitator within a time agreed locally | - Two attempts by phone are made to contact clients who have been referred or who have missed a scheduled appointment |
| - Community Facilitators will contact no-shows and if they signal that they are dropping out, Community Facilitators will administer drop-out questions | - Following the second unsuccessful attempt, a text, email or letter will be sent to the client to inform them that the service has been unable to make contact and information for re-entering the service will be provided |
| - Irish Cancer Society sends letter/email in exceptional circumstances |  |
| - If participants cannot be contacted for 2 weeks, a call will be made to the pharmacy to see if they are still using Nicotine Replacement Therapy. Pharmacist asks participant to contact Community Facilitators for a chat and to confirm that they are still in WCQ and are encouraged to attend group on a weekly basis | - Clients who cannot be contacted by phone and do not respond to the text/email/letter are documented as lost to follow-up and their file closed |
| ***Description of WCQ2 Trial Protocol:*** | |
| - Structured questions for drop outs | |
| - Trial Contact log completed weekly - RA takes CO reading in all instances | - Contact log completed at end of programme delivery |
| **Domain 7: Follow-up** | |
| *How different is the intensity of measurement and follow-up of participants in the trial and the likely follow-up in usual care?*  **Score: 2/5**  **Rationale:**  Factors in WCQ2 that reduced the PRECIS-2 score:  Follow-up much more intensive than HSE usual care.   - In WCQ2 women seen every week for up to 12 weeks - In WCQ2, CO2 can be monitored every week for women. HSE follow-ups are at 4 weeks, then at 12 weeks and 12 months. - “Passport to Quit” given to participants at start of programme which includes NRT which can be monitored. - Pharmacists also work with community facilitators. - WCQ2 includes WHO wellbeing Index – quality of life (SF12) used - Completion of social network analysis questionnaire in intervention group only. - WCQ2 interview week 12 with intervention group for €20 voucher and 6 months €20 voucher if survey completed. If quit, a saliva sample is taken. | |
| ***Description of WCQ programme (Irish Cancer Society)*** | ***Description of usual care (HSE service)*** |
| - Three follow-up time points: week 12, 6 months and 12 months (follow-up measured from programme start date) | - Three follow-up time points: 4 weeks, 12 weeks and 12 months (follow-up measured from client’s quit date) |
| - Measures taken: Carbon Monoxide corroborated smoking status, Nicotine Replacement Therapy use, behavioural support, well-being (World Health Organization -5 Wellbeing Index), participant satisfaction | - Measures taken: self-reported smoking status, may be Carbon Monoxide validated |
| - Mode of follow-up: Week 12: face-to-face, 6 months telephone 12 months telephone (self-reported smoking status)  Two attempts to contact participants by phone | - Mode of follow-up: in person, by phone, by post or email/SMS |
| ***Description of WCQ2 Trial Protocol:*** | |
| - - Measurement taken from baseline at time of consent, prior to randomisation of intervention (regardless of quit date set) | |
| - Salivary sample from those that self-report that they have quit, quality of life (SF-12) | |
| - Mode of follow-up:  - 12-weeks: The Research Assistant will arrange individual meetings with participants prior to the last group session to complete the follow-up questionnaire and provide a saliva sample (only those that quit). Qualitative interview (for intervention only) post week 12 questionnaire completion. Telephone call will suffice questionnaire completion and/or interview completion if participant unable to meet face to face. - 6-Months: Research Assistant will call the participants and make a face-to-face appointment. Those who report they quit will be asked for a saliva sample. Telephone call will suffice questionnaire completion if participant unable to meet face to face. - Voucher €20 provided to women if interview/questionnaires completed at 12 weeks - Voucher €20 provided to women if 6 months survey completed - Three attempts to contact participants by phone - Structured dropout questions | |
| - Community Facilitator diary & checklist completed |  |
| - WHO Wellbeing Index not part of trial |  |
| - Completion of Social Network Analysis questionnaire |  |
| **Domain 8: Outcome** | |
| ***T****o what extent is the trials primary outcome relevant to participants?*  **Score**: 1/5  **Rationale**: Recruitment and retention, vis a vis a pilot feasibility study, are the primary outcome for the WCQ2 trial. This is relevant to the funder (Health Research Board Ireland) but deemed explanatory from the perspective of the women wanting to quit smoking as well as community facilitators, GPs and healthcare workers referring the women. | |
| ***Description of WCQ programme (Irish Cancer Society)*** | ***Description of usual care (HSE service)*** |
| - Self-reported smoking status corroborated by Carbon Monoxide levels at week 12 | - Self-reported smoking status, may be Carbon Monoxide validated |
| - Attendance sheet |  |
| - Self-reported smoking status at 6 months & 12 months |  |
| - Case studies collected from women who have a positive story to tell about the ripple effect of quitting smoking e.g. a family member who quit as a result of them doing programme, a job change or a new life improving purchase |  |
| ***Description of WCQ2 Trial Protocol:*** | |
| - *Recruitment of four matched pairs of disadvantaged geographical districts with each pair being randomised to intervention or to usual care, with subsequent recruitment of 24-25 women who consent to participate in each of the eight districts (97 in each trial arm) during four 12-week periods.   *For noting: This is the primary outcome of the feasibility/acceptability trial. The DI outcome will be biochemically-validated smoking abstinence at end of programme (12 weeks) and at six months (secondary outcome for effectiveness trial. | |
| **Domain 9: Analysis** | |
| *To what extent are all the data included in the analysis of the primary outcome?*  **Score**: 5/5  **Rationale**: Scoring differs here compared to other Domains as scoring is based only on the trial protocol and is not derived from a comparison with usual care.  Intention to treat analysis will be used so all participants randomised to the intervention will be included in the analysis, even if they did not adhere to the intervention, attended only one session or did not attend at all. As such, all participants’ data will be used in the analysis.  There will also be a per protocol secondary analysis. | |
| ***WCQ2 Trial Protocol*** | |
| Intention to treat analysis is a comparison of the groups that includes all participants as originally allocated after randomisation.  Per protocol analysis is a comparison of the groups that includes only those participants who completed the treatment originally allocated. | |
